# Evaluation of the TrueBeam machine performance check (MPC) beam constancy checks for flattened and flattening filter‐free (FFF) photon beams

**DOI:** 10.1002/acm2.12016

**Published:** 2016-11-30

**Authors:** Michael P Barnes, Peter B Greer

**Affiliations:** ^1^ Department of Radiation Oncology Calvary Mater Hospital Newcastle NSW Australia; ^2^ School of Medical Radiation Sciences University of Newcastle Newcastle NSW Australia; ^3^ School of Mathematical and Physical Sciences University of Newcastle Newcastle NSW Australia

**Keywords:** linac quality assurance, machine performance check (MPC)

## Abstract

Machine Performance Check (MPC) is an automated and integrated image‐based tool for verification of beam and geometric performance of the TrueBeam linac. The aims of the study were to evaluate the MPC beam performance tests against current daily quality assurance (QA) methods, to compare MPC performance against more accurate monthly QA tests and to test the sensitivity of MPC to changes in beam performance. The MPC beam constancy checks test the beam output, uniformity, and beam center against the user defined baseline. MPC was run daily over a period of 5 months (n = 115) in parallel with the Daily QA3 device. Additionally, IC Profiler, in‐house EPID tests, and ion chamber measurements were performed biweekly and results presented in a form directly comparable to MPC. The sensitivity of MPC was investigated using controlled adjustments of output, beam angle, and beam position steering. Over the period, MPC output agreed with ion chamber to within 0.6%. For an output adjustment of 1.2%, MPC was found to agree with ion chamber to within 0.17%. MPC beam center was found to agree with the in‐house EPID method within 0.1 mm. A focal spot position adjustment of 0.4 mm (at isocenter) was measured with MPC beam center to within 0.01 mm. An average systematic offset of 0.5% was measured in the MPC uniformity and agreement of MPC uniformity with symmetry measurements was found to be within 0.9% for all beams. MPC uniformity detected a change in beam symmetry of 1.5% to within 0.3% and 0.9% of IC Profiler for flattened and FFF beams, respectively.

## 1 Introduction

Daily quality assurance (QA) testing of linear accelerators (linacs) is standard radiotherapy practice. In 2009, the AAPM Task Group 142 report^1^ was published to supersede the AAPM Task Group 40 for recommendations on linac QA. The TG‐142 report stipulates a daily linac QA program including testing of the photon beam output constancy.

The amorphous silicon electronic portal imaging device (EPID) has been used as a detector for linac QA measurements.^2^, ^3^, ^4^, ^5^, ^6^, ^7^, ^8^, ^9^, ^10^ EPID is well suited to a number of linac QA tests as it provides a high spatial and temporal resolution two‐dimensional digital measurement from a device that requires minimal setup time and is integrated into the linac. The latter allows for existing record and verify (R&V) databases to be used for data storage. Dose–response linearity^11^, ^12^, ^13^, ^14^ and reproducibility^15^, ^16^ of the EPID are also beneficial features for routine linac QA. A disadvantage of EPID as a detector for linac QA is the removal of the incident beam profile characteristics by the flood field correction. In addition, the high atomic number of the phosphor results in changed scattering properties compared to the equivalent depth in water^14^, ^17^ and also introduces an energy‐dependent response.^13^, ^18^, ^19^ Backscatter from the EPID positioning arm has also been a source of image nonuniformity.^20^ Solutions to some of these issues have been developed including a backscatter absorber plate between the detection panel and the positioning arm with the aS1200 EPID.

With the TrueBeam 2.0 platform, Varian released the Machine Performance Check (MPC) application. MPC is a fully integrated image‐based tool for assessing the performance of the TrueBeam critical functions. MPC tests are broken into two categories: Firstly, the beam constancy checks utilize a single MV image per beam energy without phantom in place to assess the dose, beam center, and uniformity constancy against a user‐defined baseline. Secondly, the geometric tests utilize a series of kV and 6 MV images of the IsoCal phantom situated in a specific bracket on the IGRT couch top to assess: radiation isocenter size, coincidence of MV and kV isocenters, accuracy of collimator and gantry angles, accuracy of jaw and MLC leaf positions, and accuracy of couch positioning including pitch and roll. All measurements are highly automated and the user is simply required to set up the IsoCal phantom and bracket onto the treatment couch at position H2 and to beam‐on for each required energy. For the geometric tests, the system makes all required motions automatically and beams on when all is in position. Images are automatically analyzed at the TrueBeam console and results are presented relative to the baseline. Functionality for presenting trends in results is also embedded in the MPC module and data can be exported in .csv format.

At the time of writing, there was only a single paper in the literature pertaining to evaluation of MPC. Clivio et al., 2015^21^ published work, whereby the results of MPC were compared against other more routine monthly QA techniques. In this study, both MPC and the independent QA tests were run together on 10 consecutive days. From this dataset, the time required to perform MPC was investigated, and the mean and standard deviation was calculated for both MPC and independent QA measurements and compared. The short duration of the study does not allow for any assessment of long‐term stability and there is no measure of MPC sensitivity to machine errors, both of which are acknowledged by the authors. Furthermore, the beam center constancy test is not compared against another QA method, and the QA test results are not presented in a way which allows direct comparison with MPC.

It is the aim of this study to compare the MPC beam constancy checks against both the departmental daily QA program and also to more rigorous monthly QA tests. This allows evaluation of MPC as a daily QA test device via direct comparison with current daily QA tests, and also provides an evaluation of MPC performance against more accurate monthly QA tests. The study was performed over a 5‐month time period, which allows an assessment of the MPC stability and sensitivity to drift of the linac systems being tested. The 5‐month measurement period was chosen to provide both greater than 100 MPC measurement points and also approximately 10 biweekly QA measurement points, which is the same number of measurement points used in the study by Clivio et al.^21^ Sensitivity is further examined by the use of controlled modifications to beam output and symmetry and analysis of an annual QA event, whereby beam position was required to be adjusted for a single beam. The study provides routine monthly QA results in a form that is directly comparable to the equivalent MPC test. Along with output and uniformity, the beam center constancy is also evaluated.

## 2 Methods

### 2.A Materials

All measurements in this study were performed on a single Varian (Varian Medical Systems, Palo Alto, CA, USA***)*** TrueBeam 2.0 STx linac with aS1200 EPID running both flattened and flattening filter‐free (FFF) 6 and 10 MV beams. Electron beams were not enabled on the linac. The aS1200 EPID utilizes a 43 × 43 cm^2^ panel with backscatter absorber plate between the detection panel and positioning arm. The detector matrix is 1280 × 1280 with a smaller 1190 × 1190 pixel region employed for Dosimetry (integrated) imaging mode providing a 0.34 mm resolution.

#### 2.A.1 MPC beam constancy checks

MPC is a closed system with minimal input from the user. The only parameters the user can adjust are the list of beams to be tested, the frequency of tests, and which measurement is to be used as baseline. The individual tests, reported results, and thresholds are not able to be modified by the user. For the MPC beam constancy checks, the IsoCal phantom is retracted and the EPID set to 150 cm source‐detector distance (SDD). Gantry and collimator are set to 0 degrees and an 18 × 18 cm^2^ jaw defined field is used. Forty Monitor Units (MU) are delivered per beam energy using two distinct dose rates to incorporate a dose‐rate constancy element into the testing. The raw (i.e., not flood field corrected) integrated images are analyzed. An up‐to‐date pixel correction map is required and from each image, comparison to the user‐defined baseline image is used to determine changes in output, positioning of the beam center, and beam uniformity.

For the output measurement, the mean signal from a central region‐of‐interest (ROI) of 13.3 × 13.3 cm^2^ is compared to the baseline. The use of this central ROI is to remove the effect of jaw positioning on the measurement. The output check threshold is set at ± 1.0%.

The same 13.3 × 13.3 cm^2^ ROI is used for the uniformity constancy check. In this case, the ratio of the image ROI values to the baseline image ROI values is first calculated. A high‐frequency filter is applied to remove high‐frequency noise from the image and the result presented is the variation in the two pixels with the highest and lowest ratio from the baseline.

(1) %UniformityChange=100∗(max(Ratio(x,y))−min(Ratio(x,y)))

Only a single result is presented for uniformity, so it is unclear to the user whether a measured change in uniformity relates to the inplane or crossplane direction of the beam. The uniformity threshold is set at ± 2.0%.

For the beam center constancy check, the 18 × 18 cm^2^ field edges are detected. From this, the position of the center of the beam can be determined and for each QA image, the position of this beam center is compared to the baseline position. Once again, a single result is presented and hence is not broken down into inplane and crossplane directions. The beam center constancy threshold is set to ± 0.5 mm. The linac parameters that influence each of the beam constancy check results are presented in (Table 1).

**Table 1:** Linac parameters that influence the MPC beam constancy checks

Check	Linac parameters
Output	Linac output, EPID response
Uniformity	Beam steering, beam spectrum, EPID relative pixel response
Beam center	Jaw positions, EPID detector position, beam focal spot position

#### 2.A.2 Routine quality assurance methods

The Sun Nuclear Daily QA3 device (QA3) (Sun Nuclear Corporation, Melbourne, FL, USA) is used by the department for daily linac QA. In this study, QA3 dose, symmetry, and beam position tests are compared to MPC. A Farmer type ionization chamber is compared to MPC dose and the Sun Nuclear IC Profiler is used to measure beam symmetry and focal spot position. Finally, an in‐house EPID‐based QA program is used to measure beam position, dose output constancy, jaw position, and symmetry constancy.

##### Sun Nuclear Daily QA3

The Sun Nuclear Daily QA3 model 1093 running software version 2.4.1.2 is the current device used for daily QA beam constancy checks in our department. After alignment to cross hairs or lasers, data are acquired from a single 20 × 20 cm^2^ field at 100 cm source to surface distance (SSD) and is compared to baseline. For this project, it is the dose constancy, symmetry, and beam position results that are of interest.

##### Farmer type ionization chamber

An IBA FC‐65G 0.6 cc Farmer type chamber at 10 cm depth in solid water phantom at 100 cm SSD was used as the standard for output measurement. The chamber response was traced to the secondary standards laboratory, and response constancy checks using a Strontium source were performed quarterly to ensure consistent chamber response.

##### Sun nuclear IC Profiler

The Sun Nuclear IC Profiler is a 2D ion chamber array specifically designed for beam symmetry measurements. The IC Profiler can be attached to the collimator via a gantry mount and utilizes linear arrays of ion chambers. The IC Profiler has been previously characterized by Simon et al., 2010.^22^ Besides flatness and symmetry, the IC Profiler also provides a beam center measurement. From the measured profile, the beam center is calculated as the midpoint between the 50% isodoses. When performed with 180 degree collimator rotation, the beam center measure can be used to determine the beams focal spot position.

##### In‐house EPID‐based quality assurance

The department has maintained an in‐house EPID‐based linac QA program for approximately 10 years^4^, ^5^, ^8^ and the program has been successfully migrated onto the TrueBeam linac. This program enables a unique comparison with MPC as the EPID is the common detector for both methods. Of interest to this study are the in‐house check of EPID center pixel, the dose constancy, and the profile constancy in terms of both jaw positioning and for uniformity comparison. The in‐house EPID linac QA program utilizes the EPID in integrated mode with detector at 100 cm SDD. Results are generally relative to baseline and a number of beam configurations are used. Images are analyzed using in‐house developed MATLAB scripts (The Mathworks Inc., Natick, MA, USA). The first step in the process is to image a 10 × 10 cm^2^ field at both collimator 90 and 270 degrees. From these fields, the field edges are detected and the center of the field in both planes is determined with submillimeter accuracy in EPID pixel coordinates. By averaging the collimator 90 and 270 degree images, the resulting center pixel position is dependent only on the EPID panel positioning and the focal spot position of the beam. Because of the collimator rotation, the effect of jaw positioning is removed. The measured center pixel is used as the beam central axis for reference in subsequent tests in the program.

The same 10 × 10 cm^2^ field at collimator 90 degrees is also used to assess dose constancy. A 9 × 9 pixel ROI is generated in the center of the field from which the mean integrated pixel value is recorded. This provides a measure that combines the output of the linac and any potential drift in the EPID response.

The in‐house EPID‐based linac QA uses a 20 × 20 cm^2^ field to assess profile constancy. From the same data, the accuracy of the symmetric jaw positions can also be ascertained. Profiles in both planes are extracted across the field. The coordinate system is normalized to the beam central axis from the center pixel measurement. By comparing the position of the 50% isodose for each jaw to central axis, the accuracy of the jaw positioning is measured. The profile is then compared to the baseline profile (centered based upon the baseline center pixel measurement) to determine changes in flatness and symmetry. Absolute flatness and symmetry cannot be measured as the flood field correction removes the beam horns and any asymmetry present in the beam at the time. However, for the QA program, the flood field calibration is performed immediately prior to acquisition of the baseline images which are taken immediately after the beam is steered to best achievable symmetry. This means that each time the flood field is updated, the beam needs first to be steered and afterwards QA baselines reset. The experience of the department is that in the absence of an EPID fault, flood fields need only be updated annually.

### 2.B Measurement methods

The MPC baselines were set for each beam energy. This was done following output calibration of the linac based upon monthly ion chamber readings and following beam steering based upon IC Profiler measurements. Measurements from all of the routine monthly QA methods were taken in the same session and also set as baselines. For every subsequent treatment day for the next 5 months, MPC was performed alongside QA3. Additionally, the routine monthly QA tests as outlined in Table 2 were performed on a biweekly basis. MPC results were compared both to QA3 and routine monthly QA tests over the 5‐month period. MPC short‐term repeatability was assessed by performing five successive measurements on two different days and calculating the standard deviation. The detector was not moved each day between measurements.

**Table 2:** The routine QA tests and the variables that feed into the result listed beside the MPC check they are compared to

MPC	Routine QA	
	Test	Variables
Output	QA3 dose	Linac output, QA3 response constancy
	Farmer ion chamber	Linac output, chamber response constancy
	EPID dose constancy	Linac output, EPID dose–response constancy
Beam center	QA3 X and Y shift	Cross wire alignment, setup accuracy, jaw calibration, focal spot position
	EPID center pixel	EPID detector position, focal spot position
	IC Profiler focal spot position	Focal spot position
Uniformity	QA3 combined symmetry	Setup accuracy, beam symmetry
	IC Profiler combined symmetry	Beam symmetry
	EPID symmetry constancy	Beam symmetry, EPID relative response

For the beam center constancy and uniformity constancy comparison, the routine QA results were presented in a form most directly comparable to MPC. For the beam center check, the routine QA inplane and crossplane results were both determined. The plane with the greater deviation was compared to MPC.

Using the IC Profiler, the focal spot position was calculated using its inbuilt beam center parameter and measurements from collimator 90 and 270 degrees. The sense of the collimator 270 degree beam center measurement was reversed to put both measurements in the same coordinate system. The mean beam center then represents the distance of the focal spot from center of collimator rotation. This method is independent of jaw position and IC Profiler positioning. In‐house EPID jaw position measurements were analyzed in conjunction with EPID center pixel measurements and the aforementioned IC Profiler focal spot position measurements to isolate the same three variables as the MPC beam constancy check (jaw position, EPID panel position, and focal spot position) to allow an almost direct comparison and to determine which parameter dominates the MPC measurement.

As the uniformity check gives a single result encompassing both planes then changes in symmetry in both planes will contribute to the beam uniformity result. For large fields with flattened photon beams, a change in beam angle steering primarily results in one side of the profile increasing, while the other side decreases. This simple concept provides a way in which the 1D symmetry values from the two planes can be compared to the single uniformity result from MPC. This was done by taking the routine QA symmetry change from baseline results for QA3, IC Profiler, and in‐house EPID QA calculated for both inplane and crossplane and summing the symmetry magnitudes. The result can then be directly compared to MPC uniformity to demonstrate whether the MPC uniformity can be related to symmetry. The other effect of beam steering misalignment is a change in the focal spot position. This change will result in a lateral shift of the beam and hence would be detected with the MPC beam center check.

#### 2.B.1 MPC output sensitivity

The MPC output sensitivity was evaluated in two ways. Firstly, it was determined whether the deviation of MPC output from the local standard, Farmer ion chamber readings systematically changed over the 5‐month period. Such a change would indicate a drift in the MPC response considering that the regular constancy checks using a strontium source showed no such drift in the ion chamber response. Secondly, the output of the linac was adjusted by approximately 1.2% for all energies based upon the Farmer ion chamber measurements. Three successive measurements of MPC were performed immediately before and after this adjustment and the mean measured variation was compared to the ion chamber.

#### 2.B.2 MPC beam center sensitivity to change in focal spot position

As part of routine Annual QA testing, it was discovered that for the 6 MV beam the focal spot positioning in the crossplane direction was outside departmental tolerance based upon the IC Profiler method previously described. The effect of this error was a lateral shift in the field of 0.4 mm compared to collimator rotation axes. It was thought that the QA3 field shift and MPC beam center check should be sensitive to this change so both were run before and after the adjustment and results compared to the IC Profiler measured results.

#### 2.B.3 MPC uniformity sensitivity to changes in beam angle steering

The MPC beam uniformity check was evaluated to determine whether it was sensitive to changes in beam symmetry. To evaluate this, the linac beams were directly mis‐steered by varying the angle steering based upon the symmetry measured using the IC Profiler. MPC was performed and the measured variation in uniformity was compared to the IC Profiler.

The first step in the process was to validate the IC Profiler against the standard water tank scan. An IBA CC13 chamber was used to measure inplane and crossplane dose profiles at d_max_ depth and 100 cm SSD with a 30 × 30 cm^2^ field for all four beams. The setup was replicated with the IC Profiler and the measured symmetry values from the two methods were compared using the IEC 60976 definition.^22^

Once the IC Profiler symmetry had been verified, the four beams (6 MV, 10 MV, 6FFF MV, and 10FFF MV) were steered as closely as possible to perfect symmetry (within 0.5%). MPC measurement was then performed and the baselines for each energy were reset. The beam was subsequently steered by adjusting the radial and transverse angle servo meters until 1.5% symmetry was measured on IC Profiler. This was performed individually in each of the four possible directions (+ and −, inplane, and crossplane) for each energy before being steered back to ideal. At each point, MPC was performed three times for each beam energy. Initially, the IC Profiler was set to measure symmetry based upon the detected beam center from the 50% isodoses rather than the center detector. However, it was subsequently realized from the voltage changes on the linac steering coils that the 10 MV FFF beam was steered further than the other beams. Inspection of the profiles revealed that the effect of angle steering on the profile was to shift the peak laterally. From this observation, it was decided to present the symmetry relative to the central detector rather than measured center of the beam, which is a better representation of the collimator axis of rotation. This brought the measured symmetry values in line with the changes in voltage on the steering coils, but meant that the steering of the 10 MV FFF beam was up to 4.2% rather than 1.5% as per the other three beams.

## 3 Results

### 3.A MPC beam constancy repeatability

The repeatability measurements showed that output was repeatable to within ± 0.03%, the uniformity to within ± 0.1%, and the beam center to within ± 0.04 mm. Before repeatability measurements were performed, the baselines were reset. It was assumed that the results immediately post baseline reset would come back close to zero. In the 16 measurements (four measurements for each of four beams) taken for repeatability, it was found that the beam center averaged 0.05 mm ± 0.03 (1 SD), the output averaged 0.01% ± 0.06 (1 SD), and uniformity averaged 0.50% ± 0.12 (1 SD).

### 3.B MPC output

**Figure 1:**
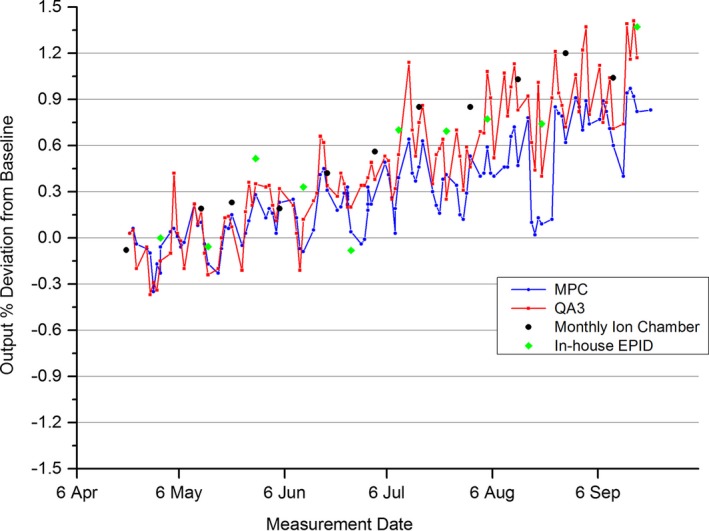
6 MV Measured output variation of the 6 MV beam over a 5‐month period as measured by MPC, ion chamber, QA3, and in‐house EPID.

The MPC measured output was plotted over a 5‐month period against the Daily QA3, biweekly ion chamber, and a biweekly in‐house developed EPID‐based dose constancy check. The results for the 6 MV beam are representative of all beams and are presented in Fig. 1.

**Figure 2:**
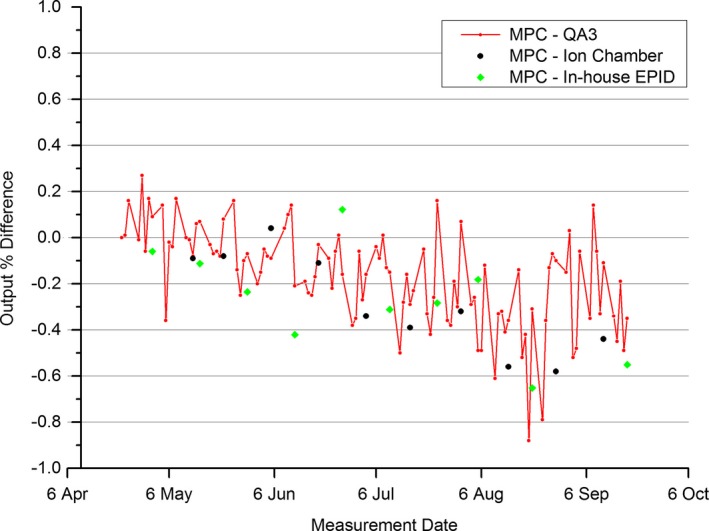
6 MV Measured output variation of the 6 MV beam MPC compared to other methods.

The results of Fig. 1 show a steady increase in output across all measurement methods. Figure 2 presents the same data as Fig. 1, but in the form of measured differences between MPC and each of the other measurement methods.

Figure 2 shows a drift downward over time of MPC against the other three methods. This indicates that the MPC response is drifting over time. When compared to the standard ion chamber over the 5‐month period, the greatest difference to MPC was measured at 0.6%. An instance occurred during the 5 months of data acquisition, where the 6 MV output measure recorded a failure of the order of 20%. This occurred immediately after a linac fault which resulted, among other things, in the EPID panel losing power and sufficient warm up time not being observed before taking a measurement. This data point was removed from the analysis in this study.

### 3.C MPC output sensitivity

The results of Fig. 1 show how the MPC output is sensitive to gradual changes over time. The result of Table 3 shows how sensitive the MPC output is to a large sudden change in output. Table 3 shows that for a Monitor Chamber output adjustment of approximately 1.2%, the MPC measured output change is in agreement with ion chamber to within absolute difference of 0.17% for all energies.

**Table 3:** Sensitivity of MPC output check to an approximate 1.2% output adjustment of the linac output

Energy (MV)	Output adjustment (% difference)		% Difference (ion chamber–MPC)
	Ion chamber	MPC	
6	−1.25	−1.27	0.02
6 FFF	−1.19	−1.36	0.17
10	−1.26	−1.34	0.08
10 FFF	−1.21	−1.35	0.14

### 3.D MPC beam center

**Figure 3:**
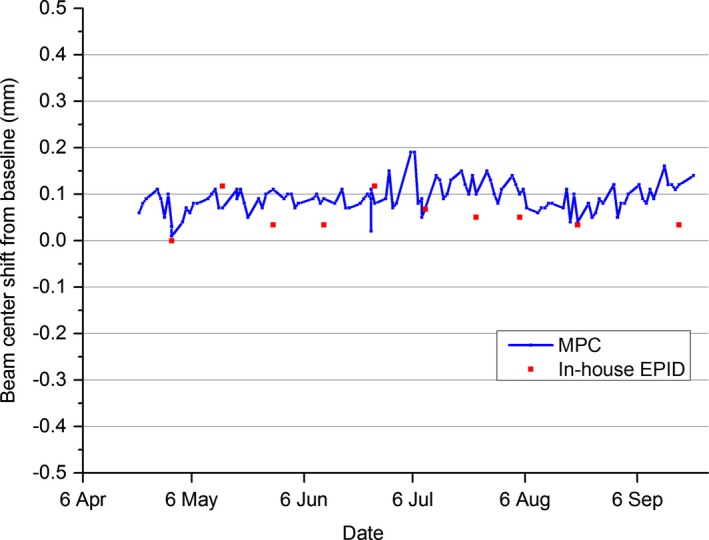
Measured MPC beam center shift for the 6 MV beam compared to the shift measured using an in‐house EPID methodology.

The results of Fig. 3 show the trend in the beam center shift for the 6 MV beam over a 5‐month period and how well the MPC measurement agrees with an in‐house developed EPID‐based method.

Over the 5‐month period, the MPC beam center showed no obvious trend. The mean difference from baseline was calculated to be 0.09 mm ± 0.03 (1 SD) (n = 115), while for the in‐house EPID method, the mean difference from baseline was found to be 0.05 mm ± 0.04 (1 SD) (n = 10). The in‐house EPID method is only performed using the 6 MV beam so this comparison is not made for the other beam energies. However, over the measurement period, the MPC mean difference from baseline and standard deviation values were calculated to be: 0.11 mm ± 0.03, 0.17 mm ± 0.05, and 0.11 mm ± 0.03 for the 10 MV, 6 MV FFF, and 10 MV FFF beams, respectively.

**Figure 4:**
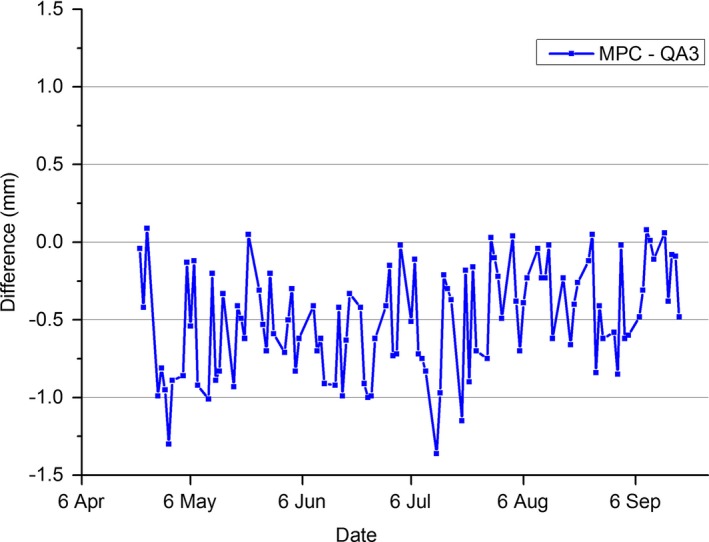
Difference in measured MPC beam center shift for the 6 MV beam compared to the shift measured using the Daily QA3 device.

The results of Fig. 4 present the absolute difference of MPC beam center compared to the maximum QA3 field shift from inplane and crossplane directions for the 6 MV beam. The mean of this data was calculated to be −0.49 mm ± 0.34 (1 SD) indicating a systematic variation in MPC when compared to QA3.

**Figure 5:**
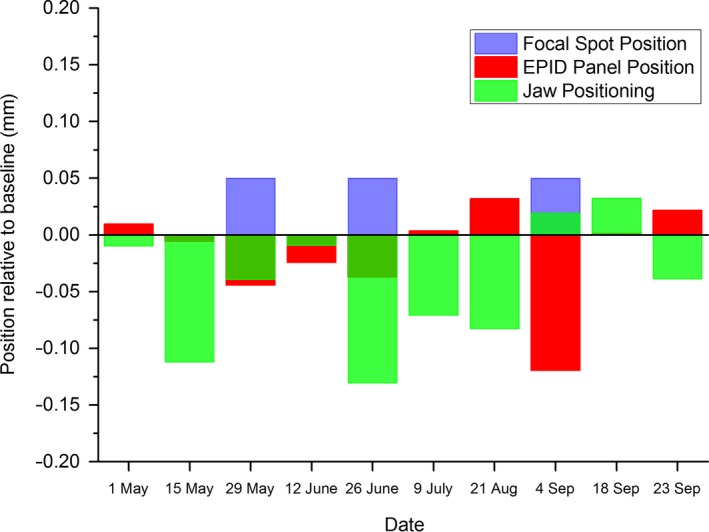
Measured changes in position from baseline for the three parameters that effect the MPC beam center measurement measured using in‐house EPID and IC Profiler. Results presented for the plane on the day which had the greatest total magnitude to allow direct comparison to MPC. The sum of the three parameters is equal to the in‐house EPID data presented in Fig. 3.

Figure 5 presents the in‐house EPID data of Fig. 3 broken into the three contributing parameters (beam focal spot position, EPID panel position, and jaw collimator position). This allows the user to note which linac parameter has greatest effect on the measurement and whether linac parameters are working in opposite directions and hence canceling each other out. Figure 5 indicates that in the majority of the measurements, it was the jaw positioning that was the greatest contributor to beam center variation.

### 3.E MPC beam center sensitivity to change in focal spot position

The results of Table 4 show how sensitive the IC Profiler focal spot position, MPC beam center, and QA3 field shift were to a change in the beam position steering. The IC Profiler measured a change of 0.40 mm, MPC measured a change of 0.41 mm, and QA3 measured a change of 0.20 mm. The standard deviation recorded indicates that the measured change for the IC Profiler and MPC were well outside measured day to day variation, while for the QA3, the measured difference was within the normal day to day variation and hence not detectable.

**Table 4:** Measured 6 MV field shift following beam focal spot position adjustment. IC Profiler focal spot position, MPC beam center, QA3 field shift

Distance (mm)	IC Profiler	MPC	QA3
1 SD	0.03	0.03	0.42
Before	0.45	0.09	0.30
After	0.05	0.50	0.10
Difference	0.40	0.41	0.20

Note: 1 SD refers to one standard deviation in measured results over the 5‐month period of measurements giving an indication of the day‐to‐day variability.

### 3.F MPC uniformity

**Figure 6:**
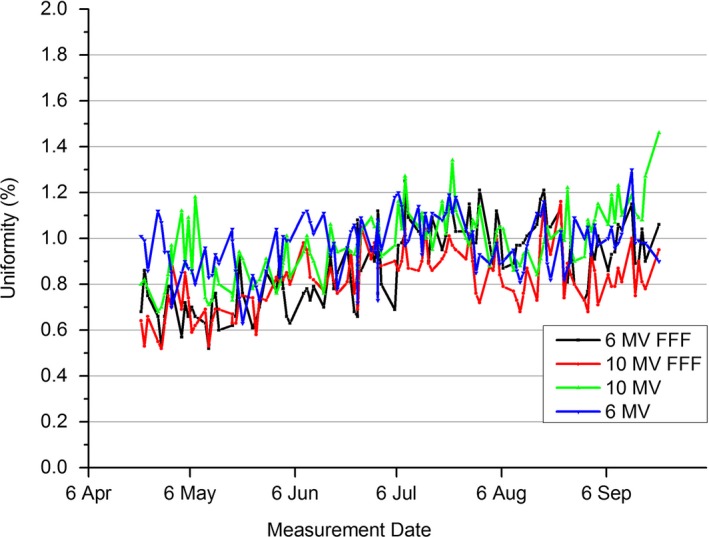
MPC uniformity results for the four beams (6 MV, 10 MV, 6 MV FFF, and 10 MV FFF) over the 5‐month measurements period.

The results of Fig. 6 present the MPC uniformity results for all four beams (6 MV, 10 MV, 6 MV FFF, and 10 MV FFF) over the 5‐month period. The plot shows similarity in measured uniformity across the four beams. For each beam, the uniformity ranges between 0.4% and 1.4%. Note that even for the first measurement point which was taken the day after the baseline was set, the measured uniformity deviated on average by 0.5% for the beams. This effect was verified in a later experiment where five uniformity measurements were performed immediately after one another and the first set as baseline. The average uniformity measured in the four successive measurements after baseline was 0.62%, 0.58%, 0.40%, and 0.39% for 6 MV, 10 MV, 6 MV FFF, and 10 MV FFF, respectively. This results in a systematic offset in the data which will affect the agreement with other methods.

**Figure 7:**
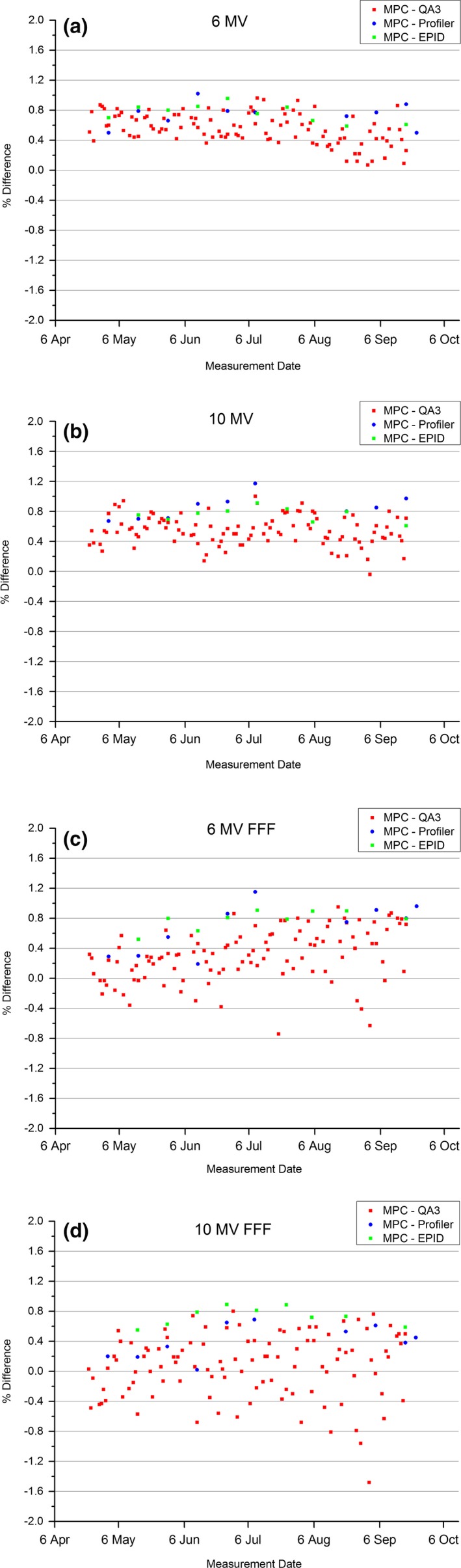
Measured MPC uniformity agreement with QA3, IC Profiler, and in‐house EPID symmetry combined from both planes. (a) 6 MV, (b) 10 MV, (c) 6 MV FFF, and (d) 10 MV FFF.

The results of Fig. 7 show how well the MPC uniformity measure compares to symmetry measurements from commonly used QA devices over a period of 5 months. The QA3, IC Profiler, and in‐house EPID symmetry measurements have been presented as inplane and crossplane added so that a meaningful comparison with MPC uniformity is made.

Figure 7 shows no overall trend, so a comparison of the mean and standard deviation of the data is presented in Table 5 for all energies. Figure 7 and Table 5 show that none of the methods agree with MPC uniformity within one standard deviation except the FFF beams using QA3; however, the MPC versus QA3 results for the FFF beams also have the largest standard deviation.

**Table 5:** Mean percentage difference between MPC uniformity and combined symmetry from routine QA methods. (Mean ± 1 SD)

Beam	MPC–QA3	MPC–Profiler	MPC–in‐house EPID
6 MV	0.53 ± 0.20	0.74 ± 0.16	0.80 ± 0.11
10 MV	0.54 ± 0.19	0.90 ± 0.20	0.76 ± 0.09
6 MV FFF	0.33 ± 0.35	0.68 ± 0.33	0.78 ± 0.13
10 MV FFF	0.08 ± 0.44	0.41 ± 0.22	0.73 ± 0.12

### 3.G MPC uniformity sensitivity to changes in beam angle steering

The symmetry measured from the IC Profiler compared to CC13 measured profiles showed agreement within absolute difference of up to 0.47%. The worst case was for the 10 MV FFF crossplane result with all other results within 0.33%.

**Figure 8:**
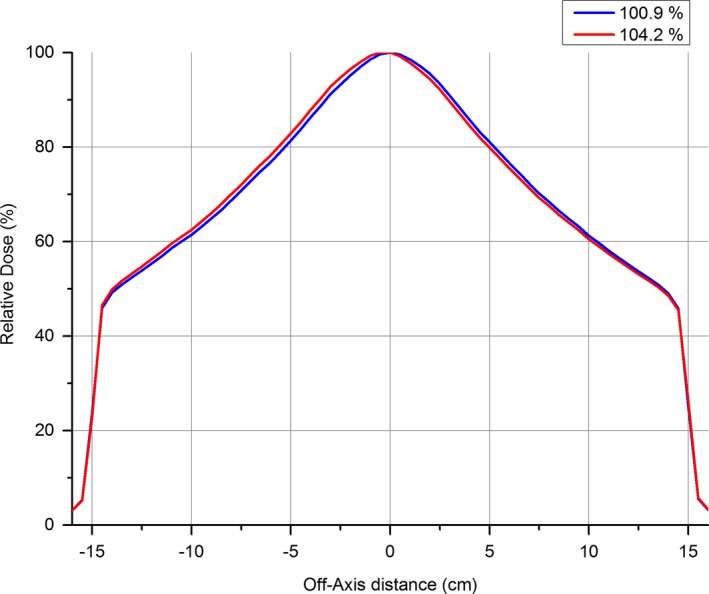
10 MV FFF inplane dose profiles with IEC symmetry of 100.9% and beam angle steered until IEC symmetry of 104.2% normalized to central chamber.

Table 6 demonstrates how sensitive the MPC uniformity check is to changes in beam angle steering. Table 6 shows that for a 1.5% change in symmetry in each of the four possible directions, the MPC uniformity agrees with the IC Profiler symmetry to within a difference of 0.30% for 6 and 10 MV flattened beams. For the 6 MV FFF beam, the difference is up to 0.93%. For the 10 MV FFF beam, the angle steering was adjusted until symmetry was measured as up to 4.2% on IC Profiler. This is a greater magnitude then the 1.5% adjustments made for the other beams. Figure 8 shows that for 10 MV FFF, the agreement between MPC uniformity and IC Profiler symmetry is within a difference of 1.4%. For both FFF beams, the measured symmetry combined from both planes is greater than the MPC uniformity suggesting that MPC uniformity is less sensitive to symmetry changes than the IC Profiler.

**Table 6:** Percentage difference between MPC uniformity and IC Profiler combined symmetry for varying beam angle steering. (MPC–IC Profiler)

Beam (MV)	0% initial	1.5% IP	−1.5% IP	1.5% CP	−1.5% CP	0% final
6	0.19	−0.11	−0.28	−0.16	−0.08	0.27
10	0.30	−0.04	0.06	−0.18	−0.29	0.04
6 FFF	−0.46	−0.88	−0.75	−0.75	−0.93	−0.59
	**0% initial**	**2.9% IP**	−**4.2% IP**	**2.6% CP**	−**2.5% CP**	**0% final**
10 FFF	−0.71	−0.22	−1.39	−1.02	−1.40	−1.31

Figure 8 shows the 10 MV FFF dose profiles measured with IC Profiler with measured symmetry at 100.9% and 104.2%, respectively, demonstrating the change in profile shape of the FFF beam due to angle steering.

## 4 Discussion

### 4.A MPC output

The divergence of MPC output from the other methods presented in Fig. 2 indicates a drift in response of MPC. The QA3 response is linked to the ion chamber by resetting of the baseline when QA3 and ion chamber results differ by more than 1%. The ion chamber response is checked with a strontium source every quarterly and no drift was detected in these checks performed before, during, and after the measurement period suggesting that the ion chamber results can be relied upon during these measurements. This suggests that it is MPC drifting in response rather than the other methods. The divergence is also apparent when comparing MPC to the in‐house EPID measure. As both of these methods utilize the EPID as the detector, the differences in the EPID acquisition would appear to be the source of the divergence. These differences include EPID calibrations including that MPC is not flood field corrected and potential difference in the dark field application, field size and ROI size differences, mode used for acquisition (in‐house EPID using treatment mode, MPC using its own MPC major mode), the varied dose rate used for MPC rather than the constant dose rate for the in‐house EPID. There is no clear evidence, which if any of these acquisition differences might be causing the divergence.

The drift detected in the MPC output suggests that to ensure accuracy of MPC output results, MPC should be compared regularly against ion chamber measurement and the MPC baseline reset if the discrepancy becomes too large. Considering the AAPM TG‐142^1^ tolerance on daily X‐ray output of 3% and the MPC output threshold of 1%, then a tolerance of 1% agreement between MPC output and ion chamber would ensure that MPC would exceed the TG‐142 daily output criteria. MPC output comparison with ion chamber has been implemented in the department on a monthly basis.

### 4.B MPC beam center

The results of Fig. 3 do not indicate a trend in the MPC beam center measurement over the 5‐month period investigated. As there was no apparent drift in the results over the period then comparison of the mean values of MPC and the in‐house EPID method is meaningful. The MPC mean was within one standard deviation of the mean in‐house EPID method indicating agreement between the two methods.

The MPC beam center mean values for all four beams indicate a small systematic offset in the data. This is also present in the in‐house EPID data. This would suggest a change in one or more of the influencing linac parameters immediately after baselines were taken. Figure 5 indicates that this is most likely dominated by a change in jaw position.

The comparison between MPC beam center and in‐house EPID is a like‐to‐like comparison as both methods utilize the same detector (EPID) and are influenced by the same linac parameters. As EPID is nonstandard for beam positioning QA, this comparison does not indicate how well MPC agrees with routine daily methods. Comparison between MPC and a routine daily beam center QA method is presented in Fig. 4, where the difference in MPC beam center is compared to the Daily QA3 device. The mean agreement was 0.49 mm ± 0.34 (1 SD). The greater variation in MPC results from QA3 compared to MPC versus the in‐house EPID method is attributed primarily to uncertainties in the QA3 setup. The QA3 is setup to the cross hairs and any positional setup errors feed directly into the beam position measurement. Over the period neither the QA3 nor MPC indicated a fail in beam position.

The results of Table 3 indicate that the MPC beam center is both sensitive and accurate to a large sudden change in focal spot position. The measured change for MPC was within 0.01 mm of IC Profiler. The magnitude of the MPC change (0.41 mm) was much greater than the measured day‐to‐day variation (0.03 mm) demonstrating that the change was detectable. The QA3 field shift was found to be insensitive to the change in focal spot position. The measured change on IC Profiler was smaller than the day‐to‐day variation of the QA3 measure suggesting that a change in this magnitude is not detectable by QA3.

The beam center results suggest that the MPC beam center method is superior to the QA3 field shift due to the sensitivity to a sudden change in focal spot position and the reduced uncertainty due to setup. AAPM TG‐142^1^ does not recommend a daily beam center test; however, the effect of change in beam position is to alter the spatial positioning of the penumbra. As clinical margins are tightened for stereotactic type treatments, the accurate spatial position of the penumbra becomes more critical so that having an accurate and sensitive daily test becomes prudent. The MPC method has been validated with an independent EPID‐based method and found to be sensitive and accurate to a large sudden change in focal spot position; it is suggested that the MPC beam center check is suitable for daily beam positioning QA. The method could be improved by taking two measurements one at collimator 90 degrees and one at 270 degrees, and the mean beam center from the two images taken as the result. This would average out the effect of jaw positioning on the measurement meaning that the measurement is only influenced by EPID panel positioning and focal spot position. Note that jaw calibration is tested as part of the MPC geometric tests so it still would be tested as part of a daily MPC QA. The obvious downside to taking the extra measurement with collimator rotation is the extra time required to rotate the collimator and deliver the beam. The authors feel that this extra time would be justified to improve the test and allow easier diagnosis of the cause of a recorded fail. An alternative is to subtract the jaw calibration variation measured in the MPC geometric tests from the beam center measurement negating the need for an extra field.

### 4.C MPC uniformity

The uniformity results of Fig. 6 demonstrate a systematic offset from the baseline present in all four beams. As the uniformity is measured by taking the ratio of the measured image against the baseline image, it would be expected that images taken immediately after the baseline would provide a result close to 0%, which may drift away from 0% over time. This is not evident in Fig. 6. Measurements taken immediately after resetting the baseline indicated an average 0.5% systematic offset in uniformity. This is unexpected and not within measurement repeatability (± 0.12% 1 SD). No explanation is provided for this offset.

The statistical disagreement between MPC uniformity and QA3, IC Profiler, and in‐house EPID measured combined symmetry as presented in Table 5 will be influenced by the systematic offset measured with MPC uniformity. If the measured offsets are subtracted from the results of Table 5, then the agreement with MPC uniformity is within 0.31%, 0.32%, and 0.38% for QA3, IC Profiler, and in‐house EPID, respectively. For the QA3 and Profiler, this is within one standard deviation.

The agreement between CC13 measured symmetry in water tank compared to IC Profiler symmetry suggest that the IC Profiler was a suitable benchmark to use to mis‐steer the beam to allow a measure of the sensitivity of the MPC uniformity to changes in beam angle steering to be made. When the beams were mis‐steered, the measured change in MPC uniformity agreed with the combined IC Profiler symmetry to within absolute difference of 0.30% for the flattened beams for every instance (Table 6). This suggests that not only is the MPC uniformity sensitive to changes in beam symmetry caused by miscalibrated beam angle but also that the magnitude accurately reflects the degree of asymmetry. However, as the MPC uniformity gives no information as to direction and is presented as a single value and hence influenced by asymmetries in both planes of the beam then it is not possible to diagnose the cause of an MPC uniformity failure. The user would have to use MPC as a daily indicator that there are no gross changes in the beam symmetry and if a fail was detected, an alternative method is required for diagnosis.

The sensitivity of the MPC uniformity to changes in beam angle steering for FFF beams does not agree with combined symmetry from IC Profiler as well as the flattened beams. The results presented in Table 6 show a systematic insensitivity of MPC compared to IC Profiler. The results for 10 MV FFF (Table 6) show even less agreement than the 6 MV FFF beam, but as explained previously, the 10 MV FFF beam had greater magnitude of steering then the other beams and this distorts the results.

The effect of beam angle steering on the FFF profiles was found to be different than the flattened beams. For flattened beams, the expected effect of a tilting of the profile was observed, while for the FFF beams, the most noticeable change was a lateral shifting of the peak. In both cases, shifting of the positioning of the penumbra was negligible. An example of this is shown in Fig. 8. This behavior may provide an explanation as to why the MPC uniformity sensitivity does not agree with combined symmetry measurements as well for FFF beams as it does for flattened beams as shown in Table 6. The combined symmetry principle works on a simplistic assumption that when beam angle steering is applied, one side of the profile decreases by a certain magnitude and the other side increases by the same magnitude. When the two magnitudes are added, the symmetry value is obtained. Using this principle, the measured asymmetry in both inplane and crossplane can be added and this combined measurement will show the expected MPC nonuniformity of the beam if the nonuniformity is caused by beam steering (asymmetry) alone (i.e., beam energy and EPID relative detector response constant). However, Fig. 8 demonstrates that the effect of beam angle steering on FFF beams is not to reduce one side of the profile by an amount which the other side increases by, but to shift the peak laterally. This behavior can explain why the results of Table 6 do not show agreement between combined symmetry and MPC uniformity for FFF beams. This demonstrates that the combined symmetry method is not suitable for comparison between symmetry measurements and uniformity and that a different method for comparison needs to be found.

The shifting of the peak for the FFF beams contributed to initial difficulties mis‐steering the 10 MV FFF beam. While the other three beams were mis‐steered by 1.5%, the 10 MV FFF beam was mis‐steered by up to 4.2%. This was because the IC Profiler was originally programmed to set central axis for IEC symmetry determination by using the average position of the 50% points on the profile. Due to the peaked nature of the 10 MV FFF dose profile for a large field then for the 30 × 30 cm field the points at 50% of central axis lie within the umbral region of the profile rather than on the penumbra. As the beam was being mis‐steered and the dominant affect was to shift the position of the peak this also shifted the 50% points and hence made the symmetry readout insensitive to the changes in beam angle steering so that when the symmetry measurement recorded 1.5% the actual symmetry using center of collimator rotation as reference was up to 4.2%. This was not a problem for the 6 MV FFF beam as for this beam at 30 × 30 cm field size, the 50% isodose lies on the penumbra. The obvious lesson is that with larger field sizes for FFF beams, the average position of the 50% isodoses is not a good indicator for determination of center of the beam.

The instance where MPC recorded a 20% output deviation for the 6 MV beam immediately after the EPID panel had power restored is a reminder that when used for dosimetry and QA, the EPID panel is a detector susceptible to fault like any other. If MPC is to be relied upon for daily linac QA, then a QA program for the imaging systems and MPC specifically is required appropriate to their use as QA detectors.

Over the 5‐month measurement period (n = 115), drift in the results was not detected in the MPC beam center or uniformity results, which allowed meaningful mean and standard deviation values to be calculated. Clivio et al.^21^ also calculated mean and standard deviations for the MPC beam center and uniformity checks although for a much smaller dataset (n = 10). Comparison between the two studies shows agreement in calculated mean values within one standard deviation of the Clivio data for all beams in terms of uniformity and for the 6 and 10 MV FFF beams in terms of beam center. The 6 and 10 MV flattened beam center mean values were within 0.07 mm.

## 5 Conclusion

The three MPC beam constancy tests have been evaluated against daily QA and monthly QA procedures over a period of 5 months. Each MPC test has also been tested for sensitivity to appropriate changes in the linac beams that they could be expected to detect. The beam output and beam center tests have been found to be at least equivalent to routine daily QA procedures and in some ways superior. A drift in MPC output was observed that suggests that regular intercomparison of MPC output with an ion chamber is required. This is now performed monthly in the department. The uniformity test was found to give a result offset from zero in measurements taken immediately after resetting the baseline. Uniformity was found to be accurate and sensitive to changes in beam symmetry for the flattened beams, but not so for the FFF beams. The different behavior of the FFF dose profile compared to the flattened beam profile with changes in angle steering is thought to be the cause. For the beam center and uniformity tests, the results of Clivio et al.^21^ have been compared with a larger dataset with general agreement observed. In our department, we have now replaced the Daily QA3 with MPC beam constancy checks for daily linac QA. It is recognized that the insensitivity of MPC to changes in FFF beam symmetry is a limitation, but this check is not required by AAPM TG‐142. It is recommended that improvements could be made to the MPC beam constancy checks by breaking the results for beam center and uniformity into inplane and crossplane, presenting uniformity in terms of flatness and symmetry constancy and modifying the beam center measurement to take out the effect of jaw positioning, which is tested in the MPC geometric tests. It was also discovered that the effect of beam angle steering on the profile shape of the FFF beams differs from the flattened beams and consideration must be given to the metric used for beam center determination when beam steering large field size FFF beams.
